# BCI-FES With Multimodal Feedback for Motor Recovery Poststroke

**DOI:** 10.3389/fnhum.2022.725715

**Published:** 2022-07-06

**Authors:** Alexander B. Remsik, Peter L. E. van Kan, Shawna Gloe, Klevest Gjini, Leroy Williams, Veena Nair, Kristin Caldera, Justin C. Williams, Vivek Prabhakaran

**Affiliations:** ^1^Department of Radiology, University of Wisconsin–Madison, Madison, WI, United States; ^2^School of Medicine and Public Health, Institute for Clinical and Translational Research, University of Wisconsin–Madison, Madison, WI, United States; ^3^Department of Kinesiology, University of Wisconsin–Madison, Madison, WI, United States; ^4^Neuroscience Training Program, University of Wisconsin–Madison, Madison, WI, United States; ^5^Department of Neurology, University of Wisconsin–Madison, Madison, WI, United States; ^6^Department of Educational Psychology, University of Wisconsin–Madison, Madison, WI, United States; ^7^Department of Orthopedics and Rehabilitation, School of Medicine and Public Health, University of Wisconsin–Madison, Madison, WI, United States; ^8^Department of Biomedical Engineering, University of Wisconsin–Madison, Madison, WI, United States; ^9^Department of Neurological Surgery, School of Medicine and Public Health, University of Wisconsin–Madison, Madison, WI, United States; ^10^Department of Psychiatry, School of Medicine and Public Health, University of Wisconsin–Madison, Madison, WI, United States; ^11^Medical Scientist Training Program, School of Medicine and Public Health, University of Wisconsin–Madison, Madison, WI, United States; ^12^Department of Psychology, University of Wisconsin–Madison, Madison, WI, United States

**Keywords:** brain-computer interface, functional electrical stimulation, stroke, motor functional recovery, closed-loop system, open-loop system, neurorehabilitation, motor recovery

## Abstract

An increasing number of research teams are investigating the efficacy of brain-computer interface (BCI)-mediated interventions for promoting motor recovery following stroke. A growing body of evidence suggests that of the various BCI designs, most effective are those that deliver functional electrical stimulation (FES) of upper extremity (UE) muscles contingent on movement intent. More specifically, BCI-FES interventions utilize algorithms that isolate motor signals—user-generated intent-to-move neural activity recorded from cerebral cortical motor areas—to drive electrical stimulation of individual muscles or muscle synergies. BCI-FES interventions aim to recover sensorimotor function of an impaired extremity by facilitating and/or inducing long-term motor learning-related neuroplastic changes in appropriate control circuitry. We developed a non-invasive, electroencephalogram (EEG)-based BCI-FES system that delivers closed-loop neural activity-triggered electrical stimulation of targeted distal muscles while providing the user with multimodal sensory feedback. This BCI-FES system consists of three components: (1) EEG acquisition and signal processing to extract real-time volitional and task-dependent neural command signals from cerebral cortical motor areas, (2) FES of muscles of the impaired hand contingent on the motor cortical neural command signals, and (3) multimodal sensory feedback associated with performance of the behavioral task, including visual information, linked activation of somatosensory afferents through intact sensorimotor circuits, and electro-tactile stimulation of the tongue. In this report, we describe device parameters and intervention protocols of our BCI-FES system which, combined with standard physical rehabilitation approaches, has proven efficacious in treating UE motor impairment in stroke survivors, regardless of level of impairment and chronicity.

## Introduction

Stroke is most often caused by a reduction or interruption of blood supply to parts of the brain resulting in sustained damage, which may produce a variety of symptoms including weakness or paralysis of an extremity. Each year, ~795,000 people experience a new or recurrent stroke in the United States (Virani et al., 2020). Approximately 610,000 of these are first attacks, and 185,000 are recurrent attacks, making stroke a leading cause of serious long-term acquired disability in the United States. Potential recovery from stroke follows an important initial timeline as recovery potential decreases the more time passes since the initial stroke. Spontaneous recovery may occur; however, natural recovery and recovery potential plateau, leaving some stroke survivors with a lifetime need for care.

Stroke-related economic burden is immense and increasing at a rapid rate. In 2014–2015, the direct and indirect cost of stroke in the United States totaled $45.5 billion (Virani et al., 2020). The estimated direct cost of stroke was $28 billion and indirect cost (lost future productivity) $17.5 billion (Virani et al., 2020). Between 2015 and 2035, total direct medical stroke-related costs are projected to increase significantly, to $94.3 billion, with much of the projected increase in costs arising from those >80 years of age (Virani et al., 2020). Stroke-related costs, therefore, are disproportionally associated with long-term care and rehabilitation. Paradoxically, long-term stroke rehabilitation is disproportionately difficult to obtain as most healthcare payers cover only a limited number of rehabilitation visits, leaving an unmet need for affordable care options beyond the standard clinical care window for patients living with acquired motor disabilities. Therefore, an urgent need exists to reduce cost of care, improve efficacy of existing poststroke rehabilitative therapies, and develop novel therapeutic approaches so as to offer stroke survivors more cost-effective and better treatment outcomes and increased functional independence.

Conventional stroke rehabilitation approaches are interdisciplinary in nature. Dominated by physical therapy (PT), often provided in combination with occupational and speech therapies, and constraint-induced movement therapy (CIMT) (Fleet et al., 2014; Kwakkel et al., 2015), the main aim of traditional therapeutic approaches is recovery of speech and improved functional use of impaired extremities in an effort to facilitate activities of daily living (ADLs) and foster survivors' functional independence, thereby enhancing quality of life. Strong evidence exists that rehabilitation approaches that promote intense, highly repetitive active functional use of the impaired limb result in the largest therapeutic benefits (Pollock et al., 2014; Veerbeek et al., 2014). Gains in movement capability that result from physical exercise, however, are mostly task-specific and restricted to the trained functions and activities. Moreover, participation in active movement training and CIMT requires sufficient residual motor capabilities, which precludes participation of severely impaired individuals, especially during the time-critical, early phases poststroke.

Clinical interest in new therapeutic approaches in which physical exercise is combined with innovative, BCI-based treatments that may induce and/or facilitate experience-dependent brain plasticity, such as transcranial direct current stimulation (tDCS) (Lindenberg et al., 2010), transcranial magnetic stimulation (TMS) (Smith and Stinear, 2016), robot-aided therapy (Babaiasl et al., 2016; Baniqued et al., 2021), virtual reality (VR) (Laver et al., 2015; Johnson et al., 2018), and other BCI-mediated interventions is growing rapidly [for recent reviews, please see (Bockbrader et al., 2018; Bai et al., 2020; Simon et al., 2021)]. BCI-mediated interventions offer the unique potential to rehabilitate motor dysfunction following brain injury, such as stroke, regardless of level of impairment or time since the injury occurred. For example, some stroke survivors retain the capability to attempt movements with their impaired extremity during all phases poststroke and, therefore, it may be prudent to guide BCI-mediated rehabilitation toward adaptive neuroplastic changes associated with BCI-induced restoration of functional capacities rather than improved physical abilities. Importantly, BCI-based treatments allow rehabilitation of stroke survivors to commence during crucial (early) time windows poststroke and would provide alternatives for more severely impaired individuals or those who have not yet regained any overt movement capacity and, therefore, are not able to benefit from traditional PT.

Despite recommendations from the 2009 workshop sponsored by the NIH Blueprint for Neuroscience Research that heralded the translation of neuroplasticity as key to developing guidelines for innovative, effective clinical therapies in rehabilitation (Cramer et al., 2011), widespread adoption of BCI-mediated therapeutic approaches clinically has not (yet) been realized, in part because of insufficient evidence supporting their effectiveness, and in part because of practical, technological, and mechanistic factors, including high equipment costs, limited portability of equipment and the need for extensive expert supervision (Baniqued et al., 2021; Simon et al., 2021). In order for more widespread use clinically, BCI-mediated interventions must not only provide high quality rehabilitation, but they must also be evidence-based, cost-effective, user-friendly, and they must be able to actively engage both patients and caregivers while, ultimately, be adaptable for home use (Remsik et al., 2016; Simon et al., 2021).

With regard to the above list of requirements for wide-spread adoption of BCI-mediated therapeutic approaches, recent meta-analyses and reviews have highlighted EEG-based BCIs as most promising in the rehabilitation of stroke survivors (Cervera et al., 2018; Bai et al., 2020; Simon et al., 2021). Moreover, BCI paradigms utilizing FES and/or attempted voluntary movements of the impaired extremity are most effective in the rehabilitation of upper extremity (UE) motor function poststroke (Ackerley et al., 2007, 2011, 2014; Ramos-Murguialday et al., 2013; Jang et al., 2016; Biasiucci et al., 2018; Cervera et al., 2018; Nishimoto et al., 2018; Remsik et al., 2018; Tabernig et al., 2018; Bai et al., 2020) because they may induce and/or facilitate neuroplastic changes that directly link movement intent with muscle contraction (Pundik et al., 2015; Bai et al., 2020; Simon et al., 2021).

Closed-loop, EEG-based BCIs employ multimodal sensory feedback in order to provide a non-invasive neural interface that is used therapeutically to substitute or augment native neuromuscular outputs by translating user-controlled neural activity into functionally relevant and therapeutically viable command signals. More specifically, user-generated unique and measurable modulations in sensorimotor rhythms (SMRs) (i.e., event-related synchronization, ERS and/or event-related desynchronization, ERD), extracted from EEG activity associated with movement intent during voluntary real, attempted, and/or imagined movements (Wilson et al., 2009; Nam et al., 2011) are translated into external command signals which, in turn, are used to control movement of a virtual cursor (e.g., ball) on a screen (Wolpaw et al., 1991; Schalk et al., 2004, 2008; Wilson et al., 2009) or functional electrical stimulation (FES) of specifically targeted muscles or muscle synergies (De Marchis et al., 2016). Furthermore, by monitoring multimodal sensory feedback (e.g., vision of the ball on the screen, somatosensory feedback associated with FES-induced movements, etc.), BCI users are able to learn through consequence how to adjust modulations in their SMRs to improve and fine-tune command signals.

**Figure 1:**
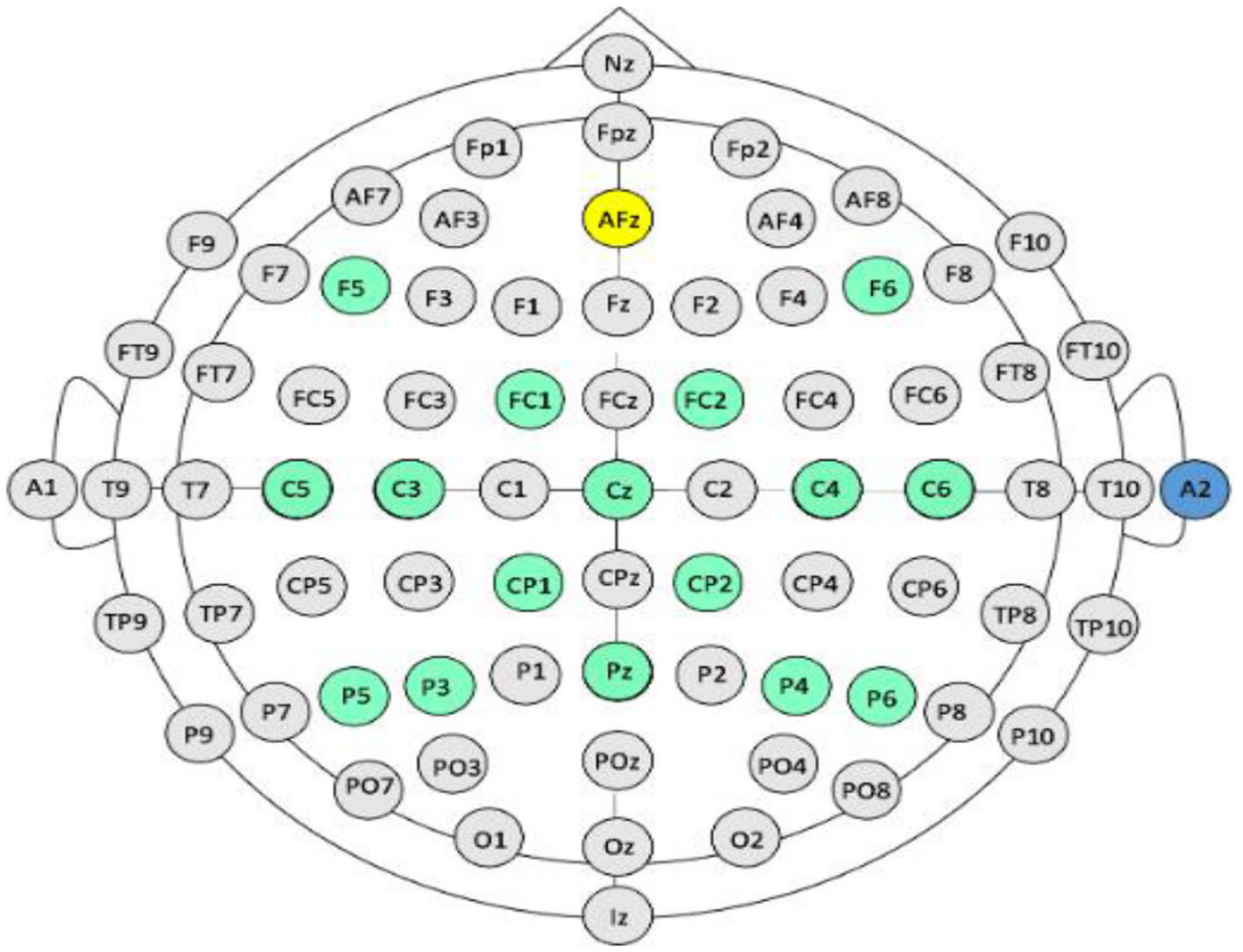
EEG electrode arrangement. International 10-20 electrode array. Yellow circle denotes ground, blue circle denotes reference electrode (on the right ear), green circles denote electrodes used from the array by the BCI.

In this report, we present device parameters and intervention protocols of our closed-loop, EEG-based BCI-FES system which, combined with standard physical rehabilitation approaches, has been validated and proven efficacious in the rehabilitation of UE motor function poststroke in our ongoing cross-over controlled clinical trial (ClinicalTrials.gov study ID NCT02098265) (Young et al., 2014a, 2015; Remsik et al., 2018, 2019, 2021). This BCI-FES system elicits positive changes in the primary outcome measure (ARAT score: Arm Reach Action Test) (Lyle, 1981) as well as beneficial physiological changes in secondary outcome measures of neural activity (e.g., Mu ERD) (Remsik et al., 2018, 2019, 2021). The system's efficacy relies on specific targeting of neuromuscular activity contingent on intent-to-move neural signals, recorded with scalp electrodes overlying cerebral cortical sensorimotor areas (Figure 1), as well as concurrent delivery of multimodal sensory feedback through implementation of a chain of straightforward operating procedures described in this report. The scalp EEG signals provide an efficient and practical way to extract, in real-time, the relevant control features, and to deliver the desired feedback to the patients as part of an interactive and closed-loop neural activity-triggered application. We also present illustrative intervention data from three stroke survivors for the purpose of illustrating the utility of this BCI-FES design in rehabilitation at various levels of impairment and chronicity. The present BCI-FES protocol, integrated with standard rehabilitation approaches, may provide a substantial improvement toward sensorimotor functional recovery of the impaired extremity in stroke survivors (Remsik et al., 2018, 2019, 2021).

## Materials and Equipment

### The Multimodal BCI-FES

**Figure 2:**
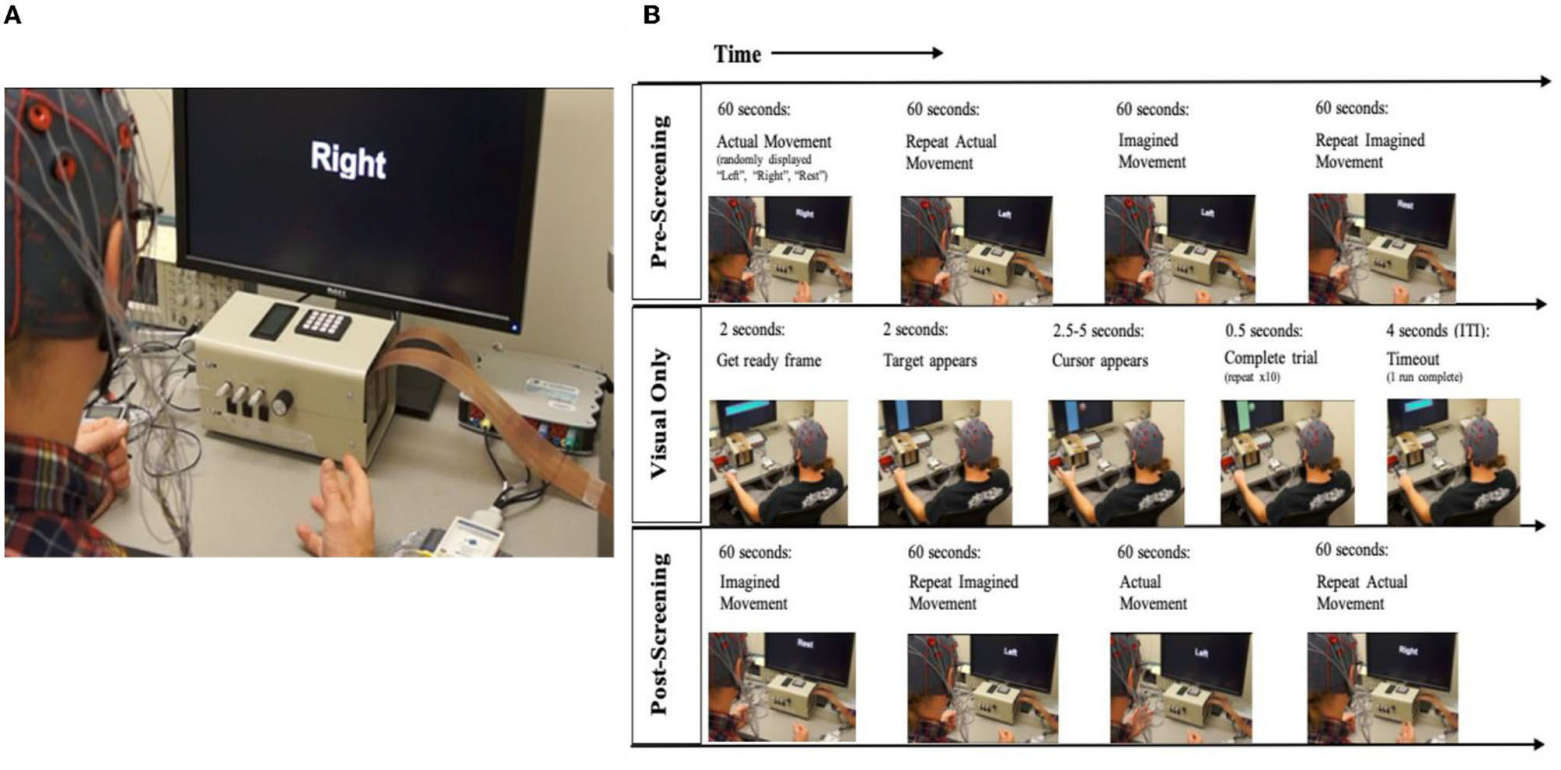
BCI setup and task block design. **(A)** Participant set up with BCI interface for open-loop trials. Setup includes monitor, EEG cap, and amplifier. **(B)** Session and block design: Every session starts with an open-loop condition, followed by the intervention (closed-loop) condition which is followed by a repeat of the open-loop condition. Open-Loop, Participants are notified that the run will begin. First the cue appears on the screen with corresponding auditory instruction for the open-loop screening condition; Closed-loop, The target appears on one side of the monitor, followed by the cursor ball in the closed-loop. Once the participant guides the ball into the target, the trial is complete.

**Figure 3:**
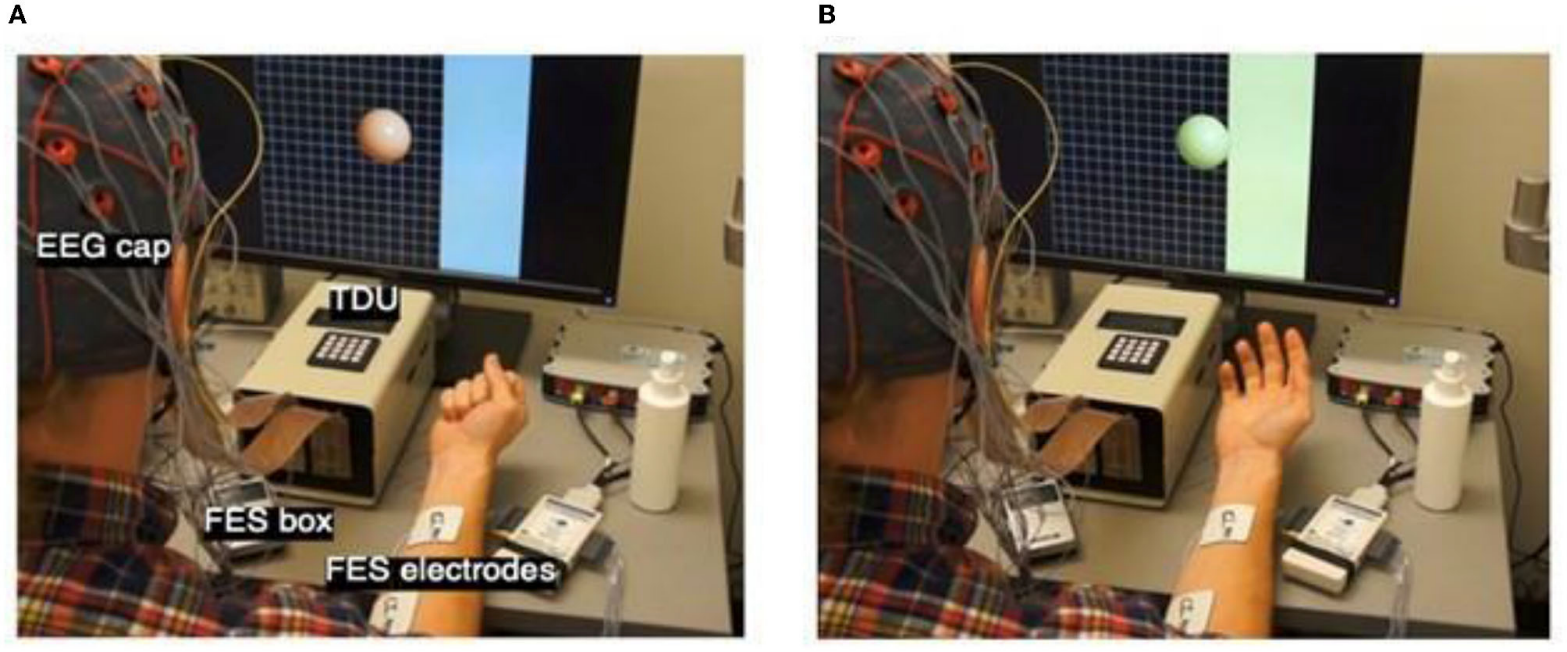
Intervention Setup and Cursor Ball Display. **(A)** Cursor appears in the middle of the screen following target presentation on one side or the other. The target is represented by the blue strip on one side of the monitor. EEG cap, FES box, FES electrodes, and TDU box are labeled to show device setup. **(B)** Cursor ball moves toward the target as cued by EEG-recorded intent-to-move brain signals. If the target is not hit in the maximum time allowed (e.g., 2.5–5 s) the trial is aborted. If the user moves the cursor into the target, the trial is a success. There are 10 trials in one run. Following 10 runs of visual stimulus only, FES is added, and 10 trials of BCI+FES later, the TDU adjuvant is included.

A conventional EEG-based BCI system presents the user with a visual display that represents modulation in SMRs related to movement intent (Pfurtscheller and Berghold, 1989; Wolpaw et al., 1991; Pfurtscheller and Lopes da Silva, 1999; Leuthardt et al., 2004; Schalk et al., 2004; Pfurtscheller et al., 2005; Daly and Wolpaw, 2008; Young et al., 2014). The BCI-FES system design presented here extends this standard paradigm by presenting the user with a virtual environment in which goal-directed motor learning is reinforced explicitly. The BCI-FES design also allows for FES-induced UE movement facilitation contingent on the cerebral cortical motor signals associated with movement intent, and for multimodal sensory feedback (e.g., visual, electro-tactile, and somatosensory) (Figures 2, 3).

#### EEG Cap Configuration & Signal Acquisition

EEG electrodes are positioned on the scalp according to the standard 10–20 system, grounded to Fpz, and referenced to an electrode placed on the back of the participant's right ear. Signals from the C3, C4, and Cz electrodes, overlying the sensorimotor cortices, are recorded in every session and are used to drive horizontal cursor movement (Schalk et al., 2004, 2008; Wilson et al., 2009) (Figure 3). EEG activity is recorded from 16 locations with sintered Ag/AgCl active electrodes using a sensor cap attached to a 16-channel bipolar recording system (g.LADYbird-g.GAMMAcap, Guger Technologies, Graz, Austria) (Figure 1). Electrode signals are amplified (g.USBamp, Guger Technologies, Graz, Austria) and digitized by 16 independent 24-bit A/D converters at 38.4 kHz per channel. EEG activity is sampled at 256 Hz, using a 0.1–100 Hz band-pass filter, and a 58–62 Hz notch filter.

#### Signal Processing

Signal acquisition, online signal processing, and behavioral task (cursor movement and virtual targets) are controlled using custom software developed on the BCI2000 platform (Schalk et al., 2004). Following basic filtering, the signal enters into a spectral estimator which computes a continually updated estimate of the spectrum of its input data. For each updated computation, the module uses a 0.5 s window of past data and applies an autoregressive (AR) algorithm to estimate spectral amplitude. The AR algorithm computes an autoregressive model of its input data using the maximum entropy method (Marple and Carey, 1989) and outputs an estimated power spectrum collected into bins. Bins are of 2 Hz width each with the center of the first bin being 0 Hz and the center of the last bin being 40 Hz. This results in 21 bins, with the first bin covering the DC range −1 to +1 Hz (which due to symmetry of the transfer function is twice the integral from 0 to 1 Hz) and the last bin covering 39–41 Hz.

Results of the spectral estimator are used in a linear classifier through a process of feature extraction and translation. The linear classifier computes a projection of a high-dimensional signal feature into a low-dimensional classification space. In our implementation, spectrum amplitudes from C3, and C4 at both 8 and 18 Hz, are translated into the one dimension of the classification space. The classifier output enters a normalization transformation of the form: *output* = *(input - o)g*, in which “*o”* is the normalizer offset value, and “*g”* is the normalizer gain. Adjusting the offsets for bias of cursor movement in the right or left direction, and gain, controls the speed at which the cursor moves. In essence, the classifier output undergoes a normalization transformation, and is then used as a control signal that specifies one-dimensional horizontal cursor movement in the user application module (Wilson et al., 2009).

#### User Application (Visual Presentation)

Following normalization, the control signal is passed to the user application. Throughout the BCI design the user-generated modulation in SMRs is time-locked to the FES and/or output of the tongue-display unit (TDU) (Kaczmarek, 2011; Wilson et al., 2012) and the visual display presentation. Recognition of attempted right-hand and left-hand movements results in concordant horizontal cursor movement in right and left directions, respectively (Wilson et al., 2009). Cursor and TDU parameters may be updated once per block of data acquisition. Data is acquired at 256 Hz, and 12 samples compose a single block. This means that the user application is updated at a frequency of 21.3 Hz or every 46.8 ms.

#### Functional Electrical Stimulation (FES)

FES of the UE (Popovic et al., 2002a,b, 2004; Peckham and Knutson, 2005; Ragnarsson, 2008; Page et al., 2009; Takahashi et al., 2012; Howlett et al., 2015; McCabe et al., 2015; Vafadar et al., 2015; De Marchis et al., 2016; Jang et al., 2016; Kim et al., 2016; Biasiucci et al., 2018; Tabernig et al., 2018; Annetta et al., 2019; Wilson et al., 2019), an established means for treating neuromuscular treatment following central nervous system (CNS) injury, is delivered in this design through a pair of square electrodes up to 2″ × 2″ in size (Figure 3), placed securely on the affected forearm using highly conductive electrolyte spray. Stimuli are produced by a LG-7500 Digital muscle Stimulator (LGMedSupply, Cherry Hill, NJ, USA). Commercially available stimulus isolation units ensure clean, opto-electrical isolation. The FES electrodes are placed superficial to the flexor digitorum superficialis muscle in order to facilitate repeated whole-hand grasping (i.e., hand and finger flexion) or superficial to extensor digitorum communis in order to facilitate repeated whole-hand opening (i.e., hand and finger extension), according to participant preference at individual BCI-FES sessions. The FES is computer-controlled using an Arduino Uno R3 microcontroller board (Adafruit Industries, New York, NY, USA) and a simple reed relay circuit. FES amplitude is set to elicit observable muscle contractions (e.g., whole-hand grasping or extension) without pain to the user. The pulse rate of the stimulation is 60 Hz, in order to produce tetanic contraction of the muscles, and the pulse width is 150 μs. Stimulation intensity is initially set to zero and is adjusted in steps of 0.5 mA, unless the stimulation becomes uncomfortable for the participant. In the event of discomfort, the stimulation intensity is returned to the nearest previous level not producing discomfort. The device is never set to deliver an output >50 mA.

#### Tongue-Display Unit (TDU)

The TDU (Figure 3A), has been described in detail previously (Kaczmarek, 2011; Wilson et al., 2012). The TDU is battery-operated and generates patterned, low-voltage stimulation to a 12 × 12 electrode array that is positioned on the anterior dorsal portion of the participant's tongue (Kaczmarek, 2011; Wilson et al., 2012). Similar to the FES, the TDU intensity is set, prior to any trials, to the highest level of intensity not producing discomfort in the participant. The TDU electrode grid supplements the visual cursor and target task; it aids participants with potential visual field impairments (Bach-y-Rita, 2004). When the target appears on either the left or right side of the display screen, the TDU electrode array is activated concurrently and concordantly. The stimulation persists on the side of the tongue according to target location until the user successfully drives the virtual cursor into the target area (Figure 3B). In the event of a successful attempt (i.e., cursor enters target area), the entire electrode array is activated until the trial times out. In the event of an unsuccessful attempt, in which the user is unable to drive the cursor into the target area before the trial time expires, the TDU ceases to deliver stimulation to the side of the tongue corresponding to the side of the screen where the target was presented.

Stimulation intensity may be adjusted after each run to ensure that the subject is able to perceive the stimulation and correctly interpret the target presentation without discomfort. All stimuli are presented within the participant's preferred stimulus intensity range, from sensation threshold to below maximum level, without discomfort. In case the stimulus-evoked sensation becomes aversive, stimulus intensity is reduced, or the stimulus array is removed from the subject's mouth. No data are available on the effects of long-term electro-tactile stimulation of the tongue; however, the study group has neither observed, nor reported any tissue irritation following tongue stimulation from over 200 subjects tested over a 10-year period (conducted under previous UW-Madison HS-IRB Protocols 2000-0119, 2000-0527, 2001-364, 2004-375, 2005-0187, 2005-0192, and 2007-0251).

### Multimodal BCI-FES Intervention

#### Task Schedule

The BCI tasks (Figures 2, 3) consists of an open-loop (Li et al., 2014) task and two closed-loop tasks (i.e., BCI with visual feedback only, and BCI with visual feedback & electro-tactile stimulation). The general difference between the open- and closed-loop tasks is the absence or presence, respectively, of SMR-driven feedback to the participant in the form of movement of a virtual cursor on the display screen toward a target or goal area (Schalk et al., 2008; Wilson et al., 2009) and associated electro-tactile sensory feedback. Such feedback is understood to aid participants in learning to control SMR modulation and successfully perform the task. As no feedback is given during the open-loop task, no learning is expected to occur during that condition. The open-loop task is designed as an initial assessment to establish, and train, the optimal SMR features that the participant will use to control the behavior of the SMR-driven feedback (i.e., the cursor/ball) during the closed-loop tasks.

#### Familiarization With the BCI Device and Procedures

The first BCI session aims to introduce the participant to the BCI device and protocol. During this initial session, the EEG cap, FES device, and TDU device are administered as described above. Stroke survivors may present with a myriad of cognitive, affective, and physical impairments (Tsao et al., 2022) and out of respect for individual participants' needs and abilities, the researchers may allow a few runs of each BCI task condition for the purpose of introducing participants to the task requirements and feedback sensations. During these preliminary sessions, the study protocol will be faithfully administered as described. Subsequent runs in all sessions aim for all BCI task conditions to be performed consistent with protocol demands.

#### Participant Criteria

Participants are individuals with motor impairments due to stroke, regardless of stroke severity, stroke chronicity (i.e., time since injury), or gender. The effectiveness of the present BCI-FES intervention in the rehabilitation of motor impairments poststroke has been validated as part of on-going clinical trial NCT02098265, in which stroke survivors participated in 9–15 BCI-FES intervention sessions (2–3 sessions per week) lasting up to 2 h, for a maximum of 30 h of intervention. Participants also contributed to behavioral testing prior to the first BCI session (i.e., Pre), at the midpoint of intervention (i.e., Mid), immediately following the last intervention session (i.e., Post), and at a 1-month, post-intervention follow-up (i.e., Follow-up) (Table 1).

**Table 1:** Motor capacity at baseline (Pre), middle of intervention (Mid), at completion of intervention (Post), one month after completion of intervention (Follow-up), and calculations of change from baseline to the two endpoints, Post and Follow-up, respectively.

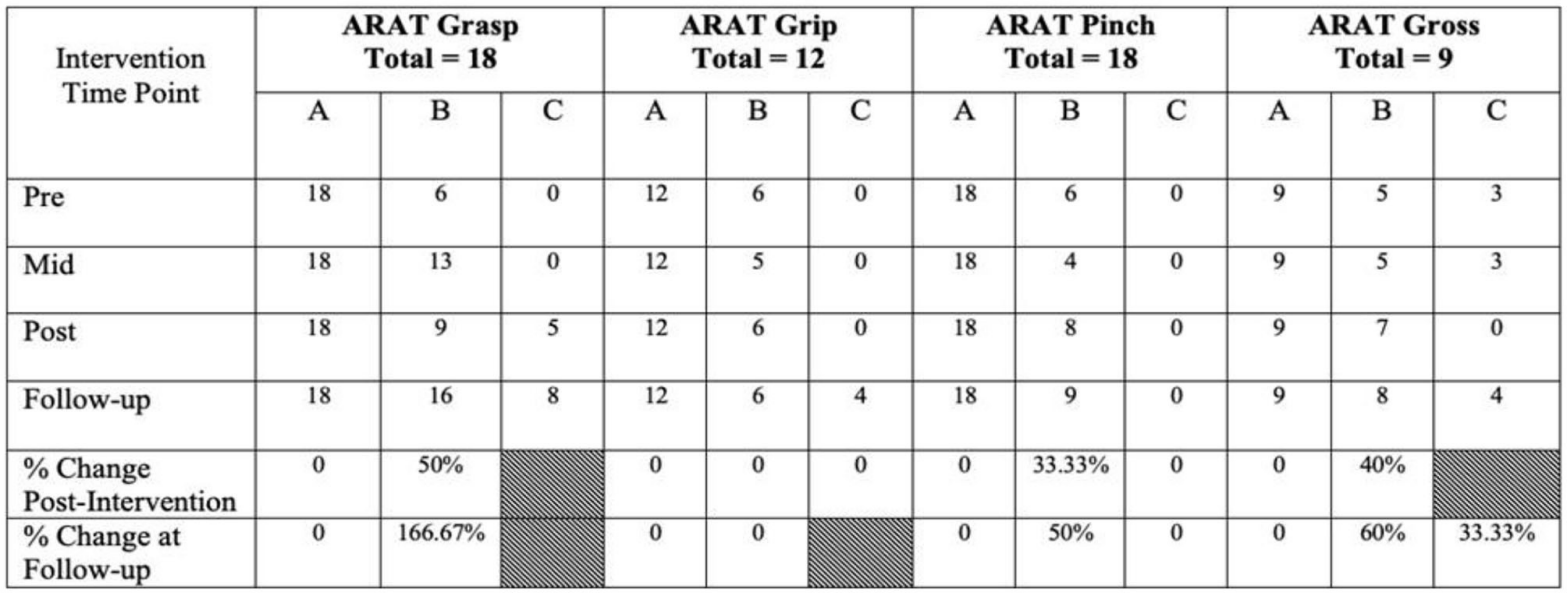

*(A,B,C correspond to participant A, B, C.) Participant ARAT subdomain scores. The maximum score for ARAT Grasp is 18; ARAT Grip maximum score is 12; ARAT Pinch maximum score is 18; and ARAT Gross maximum score is 9, for a total of 57. These data are representative of exemplar BCI-FES participants and is not intended as evidence of efficacy of this device in these participants*.

## Methods

### Setup

The EEG cap must be positioned on the user's scalp such that the electrode locations correspond with those specified by the 10–20 international system (Figure 1). All 16 electrodes used must record electrophysiological signals with optimal signal-to-noise ratios (Wilson et al., 2009).

### Protocol

#### Open-Loop Screening Task

A session begins with an open-loop hand movement assessment task, in which no performance feedback is given. The first two runs of the pre-intervention screening phase incorporate “actual, attempted” hand movements (Ackerley et al., 2011, 2014) in response to written cues displayed on the computer screen, and corresponding verbal instructions (i.e., Left, Right, Rest) (Figure 2). The last two runs of the pre-intervention screening phase incorporate “imagined” hand movements in response to the same written cues and corresponding verbal instructions (Figure 2). To accommodate initial movement capacity and the nature of each participant's motor impairment, participants are instructed to execute hand movements according to their individual treatment goals and physical capabilities but are instructed to execute repeated hand grasping motions in either hand when cued. Each screening EEG data file contains 15 trials of rest, left-hand and right-hand movements (i.e., five trials for each of the three conditions), separated by an interstimulus interval of 1.5–2 s. The order of trials in a run is random. Each of the trials has a duration of 4 s (Remsik et al., 2018, 2019). Coefficients of determination (*r*-squared) are calculated in order to evaluate the spectral difference at each frequency bin between the attempted left- and right-hand movement conditions. Finally, control features are selected as the left and right channel-frequency pairs (i.e., C3–Cz & C4–Cz electrodes as shown in Figure 1) for both the Mu (8–12 Hz) and Beta (18–26 Hz) frequency bands.

#### EEG Calibration

Data recorded during the initial screening task may be analyzed using BCI2000's Offline Analysis MATLAB-based tool in order to determine the optimal SMR features for online control of the subsequent closed-loop tasks (Schalk et al., 2004; Wilson et al., 2009; Schalk and Mellinger, 2010). The channels and frequency bands chosen should be consistent with known properties of cortical SMRs associated with attempted hand movements (i.e., locations and frequencies consistent with the contralateral cerebral cortical motor areas and the corresponding electrodes (e.g., C3, C4), and centered near the Mu (8–12 Hz), and Beta (18–26 Hz) frequency bands (Pfurtscheller and Berghold, 1989; Wolpaw et al., 1991, 2002; Pfurtscheller et al., 1997; Pfurtscheller, 1999; Pfurtscheller and Lopes da Silva, 1999; McFarland et al., 2000; Neuper and Pfurtscheller, 2001; Schalk et al., 2004, 2008; Neuper et al., 2005, 2006; Daly and Wolpaw, 2008; Ackerley et al., 2011, 2014). Control features may be standardized across subjects (i.e., 8 and 22 Hz) or optimized for each individual participant at each session. This procedure is designed to determine the features that optimize subject-specific signals that are used to drive the cursor movement and deliver concurrent FES to the stroke-impaired musculature. Although selected control features may differ between participants, the common underlying principles are that the features selected are overlying sensorimotor cortices, and that they are in the expected physiological range of motor output (i.e., ~6–30 Hz) so as to ensure they represent user-driven motor signals associated with movement intent.

#### Closed-Loop Cursor & Target Task

The control features are translated into feedback (i.e., ball/cursor movement) of the subsequent BCI tasks as described by Schalk and Mellinger (Schalk et al., 2004; Schalk and Mellinger, 2010). An autoregressive spectral analysis (Marple and Carey, 1989) first estimates the spectral power of the control features. The resulting control feature signals are then put into a classification algorithm that performs a linear transformation of these signals, which are translated into the feedback behavior of the cursor on the screen, the FES adjuvant, and the TDU stimulation. The prevailing logic is that the strongest SMR features [within the prespecified Mu (8–12 Hz) and Beta (18–26 Hz) frequency bands (Pfurtscheller and Berghold, 1989; Wolpaw et al., 1991; Pfurtscheller et al., 1997; Pfurtscheller, 1999; Pfurtscheller and Lopes da Silva, 1999; McFarland et al., 2000; Neuper and Pfurtscheller, 2001; Neuper et al., 2006; Ackerley et al., 2011; Babiloni et al., 2016)] of attempted movement define the control features used for each participant in the subsequent closed-loop (i.e., Cursor Task) condition (Wilson et al., 2009).

#### Visual Feedback Only

The first ten runs of the closed-loop BCI task condition present the user with visual feedback of their modulated SMR features through a virtual ball-and-target game (i.e., closed-loop Cursor Task) (Figures 2, 3). During this task, users perform the same type of repeated attempted hand movements as in the screening task described in Open-Loop Screening Task (above). Participants learn to control the movement of the virtual ball (i.e., cursor) displayed on the computer screen by modulating their SMR activity as they perform the task. The SMR activity, related to attempted left- or right-hand movements, are translated into leftward (or rightward) ball movement (Figures 3A,B). At the start of each trial, the participant is instructed to look at the center of the blank screen. Two seconds later, a virtual target appears randomly on the left or right side of the screen. After the target is displayed for 2 s, the cursor (ball) appears in the center of the screen and the participant is instructed to move the ball toward the target by eliciting SMR modulation using attempted repeated hand movements, as described in Open-Loop Screening Task. For a trial to be considered successful, the ball must hit the target within 2.5–5 s of its appearance. If the attempt is successful, the target appears to illuminate and maintains this “reward” presentation for 0.5 s (Figure 3). If the trial is unsuccessful after the maximum time allowed (5 s), the cursor and target disappear within the subsequent 0.5 s interval. Immediately following task completion (hit or miss), an intertrial interval of 4 s commences and the presentation sequence is repeated. Each run consists of 10 trials.

#### Adjuvant Stimulus Administration

Following 10 completed runs (i.e., 100 trials) with visual feedback only, FES and TDU (tongue-display unit) (Kaczmarek, 2011; Wilson et al., 2012) are incorporated (Figures 3A,B). Driven by the modulation in SMRs generated by engagement with the virtual ball-and-target task, FES is applied to the targeted muscles of the impaired hand and electro-tactile feedback is presented through the TDU. In this way, participants can incorporate visual, electro-tactile, and proprioceptive feedback, when possible, associated with muscle activation for the purpose of modulation and monitoring of volitional movements. The ensemble of multimodal feedback serves as adjuvant stimulus to engage paretic musculature and somato-motor circuitry in improved, more natural execution of the motor plan (e.g., attempted voluntary hand flexion) and to provide enhanced multimodal performance feedback to the user. The modulation of SMR activity needed to perform the task well directly links movement intent to the facilitated muscle contraction. Rewarding this linkage *via* the cursor-and-target task is hypothesized to facilitate motor learning and potential recovery (Bach-y-Rita, 1981, 1990; Nudo et al., 1996, 2001; Nudo, 1999, 2003a,b, 2011, 2015; Nudo and Friel, 1999; Kleim et al., 2002; Schaechter et al., 2002; Rossini and Dal Forno, 2004a,b; Plautz and Nudo, 2005; Strangman et al., 2005; Cramer and Riley, 2008; Jayaram and Stinear, 2008; Murphy and Corbett, 2009; Popovic et al., 2009; Wang et al., 2010; Ackerley et al., 2011; Cramer et al., 2011; Dimyan and Cohen, 2011; Pekna et al., 2012; Takeuchi and Izumi, 2012a,b, 2013; Wolpaw, 2012; Jiang et al., 2013; Soekadar et al., 2014; Volz et al., 2014; Reinkensmeyer et al., 2016; Biasiucci et al., 2018; Mohanty et al., 2018). BCI-driven FES is only applied to muscles of the impaired limb and is delivered only and concurrently with cursor movement toward the targeted side in order to ensure that muscle stimulation never occurs while participants attempt to move the ball toward their unimpaired side.

#### Functional Electrical Stimulation

Following 10 complete runs of BCI (visual only feedback), BCI+FES trials are initiated (Figures 2, 3A,B). FES settings are adjusted at a safe and effective intensity level as described above. The appropriate muscle(s) for targeted stimulation is (are) identified and electrodes are attached accordingly. The aim is to elicit motor responses in the impaired hand that reflect whole-hand flexion or extension. If some fingers are moving more than others, the electrodes are repositioned until fingers open/close evenly when stimulated manually. With help of the participant, the appropriate level of stimulation is established that is both comfortable for the participant and produces recognizable grasping movement of the impaired hand as described in Open-Loop Screening Task.

#### Tongue Display Unit (TDU)

Following 10 complete BCI+FES runs, BCI+FES+TDU runs are initiated (Figure 3).

#### Open-Loop Exit Screening Task

Sessions end with a repetition of the open-loop screening task as described above and in Figure 2.

### Minimizing Risks

Subjects are under supervision at all times during the experiments and are easily able to communicate discomfort or a need for respite. The preferred stimulus intensity range for FES is determined by beginning with low amplitude stimulation and gradually increasing the amplitude until the participant demonstrates a motor response or indicates that their maximal comfort level has been reached, as described previously. The amplitude threshold for eliciting a motor response generally occurs well below the amplitude threshold for stimulation discomfort. The preferred range of tongue stimulation intensity is similarly specified, but rather than looking for a motor response, the maximal range is that which provides a clear sensory percept without producing discomfort in the participant. Stimulus intensity range is determined by beginning with low-amplitude stimulation and gradually increasing the amplitude until the participant indicates their maximal comfort amplitude has been reached, as described previously. It is imperative that one listens to and engages with participants to meet their needs and maintain honorable adherence to essential principles of care such as respect for individual persons, beneficence, and justice.

## Results

Clinical efficacy of the present BCI-FES intervention in the rehabilitation of motor impairments poststroke has been validated as part of on-going clinical trial NCT02098265, in which stroke survivors participated in 9–15 BCI-FES intervention sessions lasting up to 2 h, for a maximum of 30 h of intervention per participant (Young et al., 2014a, 2015; Remsik et al., 2018). We have published evidence demonstrating improvements in both objective and subjective measures of behavioral outcomes used to assess stroke-related motor impairments (Remsik et al., 2018, 2019). For example, we have reported moderate improvements in Action Research Arm Test/Fugl-Meyer scores (Remsik et al., 2018) as compared to a control group and significantly increased grip strength (Remsik et al., 2019). Moreover, we have presented neurophysiological evidence that our BCI-FES design is able to generate significant and adaptive changes in EEG activity and brain connectivity (Mazrooyisebdani et al., 2018; Mohanty et al., 2018; Remsik et al., 2019, 2021). Specifically, increases in task-related ipsilesional Mu (8–12 Hz) ERD, were significantly correlated with improvements in measurements of motor recovery and functional connectivity (Remsik et al., 2019, 2021).

**Figure 4:**
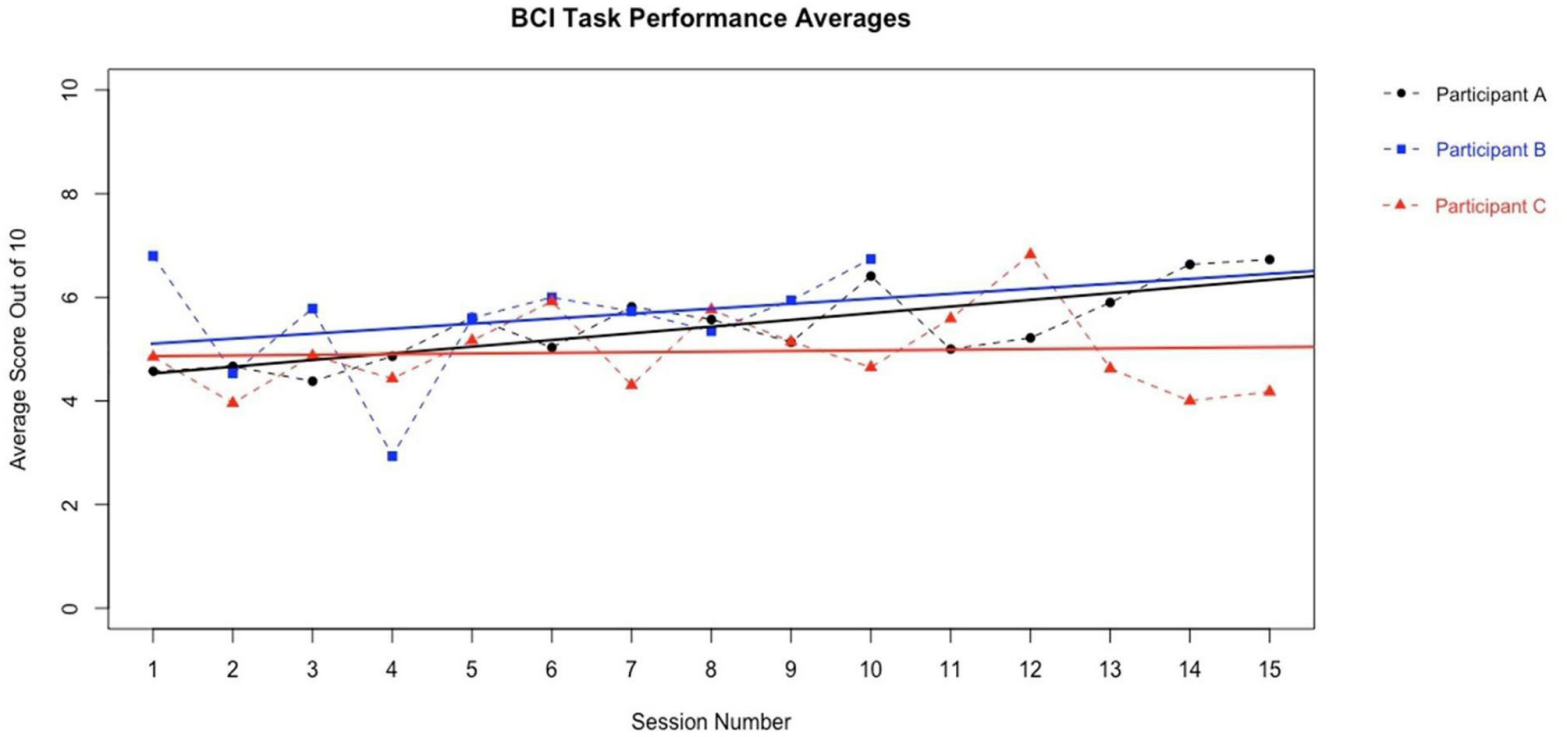
BCI Task Performance. Out of a possible 10 trials in each run, participant average BCI performance scores (i.e., how many trials a user successfully moved the cursor into the target area, x/10) for a given session across sessions are plotted with a best fit line for each participant in their own color. Y axis values represent the average performance score of all trials during the given session by a given participant. Participants improved their average BCI task performance over time. These data are representative of exemplar BCI-FES participants and is not intended as evidence of efficacy of this device in these participants.

We present example data from three participants, selected *post-hoc* from the larger cohort of participants of on-going clinical trial NCT02098265, for the specific purposes of illustrating utility and demonstrating expected outcomes of the BCI-FES intervention described in the present report—the data presented are not intended as evidence to prove the intervention's clinical efficacy. Instead, the example data illustrate outcomes of participants who differed in level of severity of stroke-induced motor impairments, from mild (Participant A), to moderate (Participant B), to severe (Participant C), and time poststroke (i.e., chronicity), from 5 months (acute, participant A), to 12 months (intermediate, Participant C) to 92 months (chronic, Participant B) (Table 2). Metrics of task performance (i.e., how many times out of 10 a participant was able to successfully move the cursor to the target) (Figure 4 and Table 3), and a primary outcome measure of functional recovery commonly used in stroke research, the Arm Research Action Test (ARAT), are included (Table 1).

**Table 2:** Participant demographics from three exemplar BCI-FES participants.

**Participant**	**Age Range**	**Pre-Stroke Handedness**	**Stroke Lesion Location**	**Impaired Hand**	**Baseline Action Research Arm Test (ARAT)**	**Impairment Severity**	**Time Since Stroke at Baseline (months)**
A	40-45	R	L Temporal	R	57	Mild	5
B	65-70	R	R PLIC Puta men	L	23	Moderate	92
C	60-65	R	R Frontal	L	3	Severe	12

*Participants A, B, and C were chosen as representative of potential BCI-FES users poststroke with varying levels of stroke-related motor impairment severity, time since stroke, stroke lesion location, concordance of stroke-impaired hand, and age. (L, left; R, right.) ARAT total scores (0 indicates severe upper extremity motor impairment, 57 indicates no measurable impairment) and baseline demographic data for each participant, A, B, and C. Chronicity is measured in months and rounded down to nearest whole month*.

**Table 3:** BCI task performance summary.

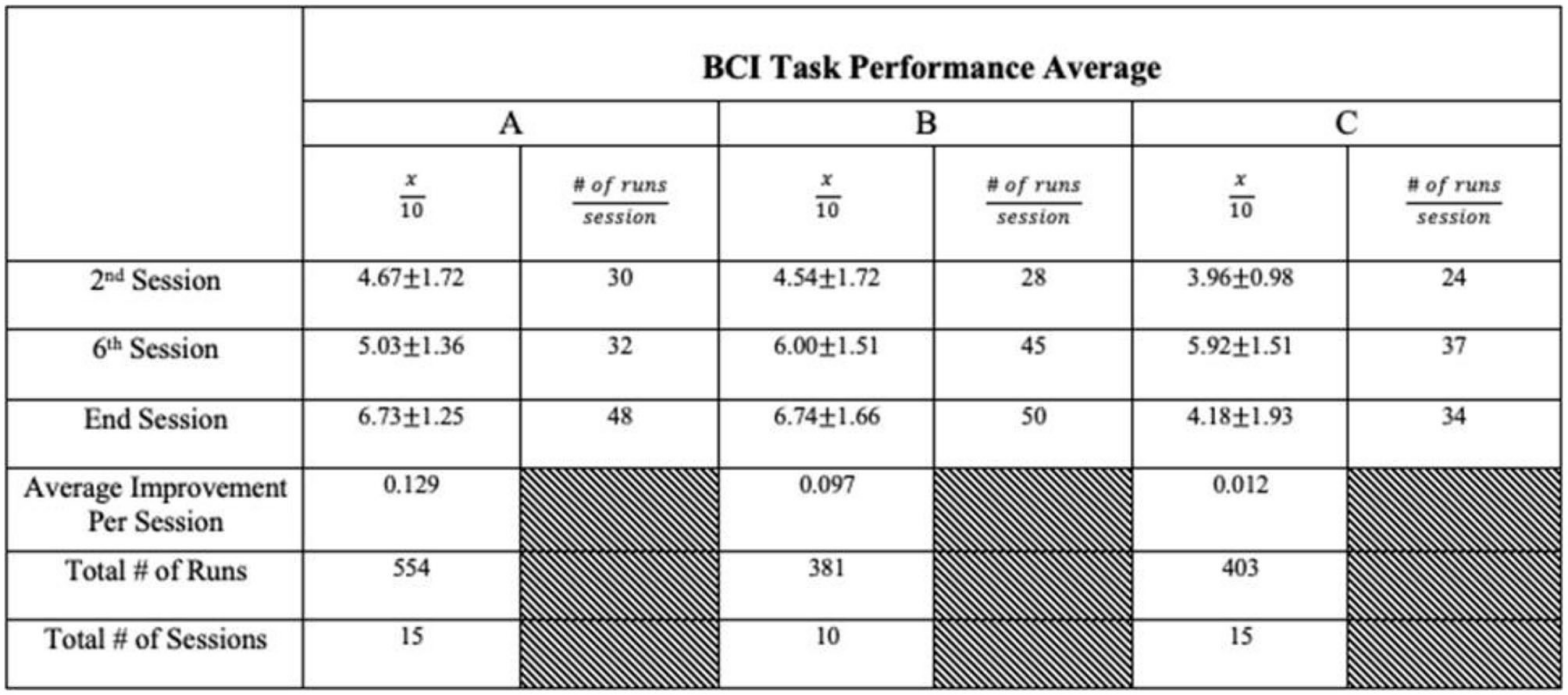

*Total change represents the difference from session 2 to the participant's final BCI intervention session. Session 1 was used partly to allow subjects to become familiar with the intervention and attention to performance was not required of the participants. X/10 indicates the average number of ‘hits' per run of the BCI task with 10 as the maximum score. Number of runs per session was determined by the number of runs completed during the allotted intervention time (2 hours); the number of runs per session for Participants A, B, and C ranged from 7 to 50. These data are representative of exemplar BCI-FES participants and is not intended as evidence of efficacy of this device in these participants*.

Table 4 summarizes a sample of validated outcome measures designed to test and quantify different functional domains that may be affected by stroke or brain injury resulting in motor loss and may be affected by this BCI-FES device design. The list is not exhaustive. In addition to behavioral and task performance measures specific to a given rehabilitation target, such as grip strength, foot-drop, spasticity, etc., it is important researchers and clinicians consider assessments of outcome measures in other domains because of the rich functional interconnectedness of the sensorimotor cortex with the rest of the brain (Simon et al., 2021).

**Table 4:** Contemporary assessments of stroke impact.

	**Description**
**Primary outcome measure**	
Action Research Arm Test (ARAT) (Lyle, 1981; Lang et al., 2006)	The ARAT is designed for evaluation of upper extremity function. This test consists of total of 19 items divided into four sections for Grasp, Grip, Pinch and Gross Movements. Item in each section is graded on a 4-point ordinal scale (zero cannot perform any part of the test, three performs normally). The maximum possible total score is 57.
**Secondary outcome measures**	
Barthel Index (Collin et al., 1988)	The Barthel Index measure a person's daily functioning (activities of daily living and mobility).
Center for Epidemiologic Studies-Depression Scale (CES-D): (Radloff, 1977)	The CES-D is a self-report scale and includes 20 items that survey mood, somatic complaints, interactions with others, and motor functioning. Responses are recorded using a 4-point Likert scale ranging from rarely (scored 0) to all of the time (scored 3), and points are summed across the 20 items to provide a total CES-D score.
DSST Mesulam and Weintraub Cancellation task for hemispatial neglect (Weintraub and Mesulam, 1985)	The Mesulam–Weintraub Cancellation task consists of four test forms utilizing structured and unstructured arrays of verbal and non-verbal stimuli. Subjects are asked to circle all of the targets they can find using different colored pencils so that after every ten targets or a specified time the participant changes pencils so that their search pattern may be identified. The targets are the letter “A” in the verbal and the symbol “” in the non-verbal arrays (~ 10 min).
Electromyography (Kauffman et al., 2021)	EMG is the recording of changes in skin voltage caused by contraction of the underlying muscles. This recording (Kauffman et al.) will be obtained using the EMG recording equipment of the BIOPAC systems (http://www.biopac.com/researchApplications.asp?Aid=41andLevel=1).
Flanker task (Eriksen and Eriksen, 1974)	Flanker task is an executive function/attention task. Subjects are presented with visual stimuli and asked to respond to the direction of a left or right pointing arrow and ignore flanking arrows that point in the opposite direction as the target arrow.
The Fugl-Meyer (FM) motor assessment (Fugl-Meyer et al., 1975)	The FM motor assessment is used to measure voluntary limb movement. It includes the upper extremity (UE) subscale (33 items; score range, 0–66) and the lower extremity (LE) subscale (17 items; score range, 0–34) for a total motor FM score of 100.1.
Geriatric Depression Scale (Yesavage et al., 1982)	Depression Screening: For subjects 65 and older, we use the Geriatric Depression Scale-15 Item. The GDS or the Mood Assessment Scale screens for depression in the elderly. The GDS taps affective and neuropsychological symptoms of depression and consists of 30 yes/no questions. For subjects younger than 65, we use the Center for Epidemiological Studies-Depression Scale. The CES-D is a self-report scale and includes 20 items that survey mood, somatic complaints, interactions with others, and motor functioning. The final score spans from 0 to 60, with a higher score indicating greater impairment (~10 min).
Hand-grip Strength (Boissy et al., 1999)	Hand grip strength is assessed with a dynamometer. Participants are asked to squeeze as hard as possible and then release. Three trials are performed with the affected and unaffected hand.
Hopkins Verbal Learning Test (HVLT) (Benedict et al., 1998)	The HVLT is a brief test of verbal learning and memory and consists of a list of 12 nouns (targets) with four words drawn from each of three semantic categories (~ 10 min).
Mini-Mental Status Examination (MMSE) (Tombaugh and McIntyre, 1992)	The MMSE is a screening tool that provides a brief, objective measure of cognitive function.
Modified Ashworth Scale (Gregson et al., 1999)	MAS assesses spasticity in wrist, elbow, and finger flexion/extension muscles, on a six-point scale (0, no increase in muscle tone to 4, limb rigid in flexion or extension).
Montreal Cognitive Assessment (MOCA) (Toglia et al., 2011)	MOCA to test subjects for cognitive impairments (~10 min).
Motor Activity Log (MAL) (Van der Lee et al., 2004)	MAL is a structured interview developed to assess the use of the more affected upper extremity in real-world daily activities. Participants are asked to rate how well (Quality of Movement) and how much (Amount of Use) they use their affected arm to accomplish 14 activities of daily living.
Modified Health Questionnaire	Modified Health Questionnaire to document the general physical health and social habits of all subjects.
The National Institute of Health stroke scale (NIHSS) (Lyden et al., 2009)	The NIHSS is a standardized method to measure the level of impairment caused by a stroke.
Nine-hole peg test (9HPT) (Mathiowetz et al., 1985; Beebe and Lang, 2009)	The participant sits at a table and is asked to take nine dowels (9 mm diameter, 32 mm long) from the tabletop and put them into 9 holes (10 mm diameter, 15 mm deep) spaced 50 mm apart on a board. The time to complete this is recorded.
Pain Scale (Wong and Baker, 1988)	Pain Scale: Participants is asked to rate their degree of pain on a scale of 0 (no pain) to 5 (in tears).
Sensory motor computerized task (Chiu et al., 2011)	Sensory motor computerized task: A computerized task testing participants speed and response time is developed in-house. The task requires participants to watch the appearance of a target on the left or right of the screen and to click the target as soon as it appears
The Short-Blessed Test (Katzman et al., 1983)	The Short-Blessed Test, a six-item test, is used as a diagnostic tool to differentiate participants with cognitive impairments from healthy controls. Subjects are asked to answer the items year and month, time of day, count backward 20-1, recite months backwards, and the memory phrase. This test is administered in addition to the MMSE, which also tests for cognitive impairment because the Short-Blessed Test is more sensitive to differences in levels of education and is quicker to administer (~3–4 min).
Span measures (Tulsky et al., 1997)	Participants recite digit span, forward and backward (measure of working memory)
Stroke Impact Scale (SIS) (Duncan et al., 1999)	The Stroke Impact Scale, or SIS, assesses changes in impairments, activities and participation following a stroke. Scores on the SIS provide an index of clinically “meaningful” change representing the change in the participant's mental and physical abilities concurrent with their performance on the verbal fluency and memory tasks. The four physical function domains (strength, hand function, ADL/IADL, and mobility) is collapsed to a physical function subscale. All domain scores range from 0 to 100 with 100 being the best.
Stroop Task (Golden et al., 2003)	Stroop task is an executive function/conflict resolution task. In this task the participant tries to name the color of the ink in which a word is printed when the word itself is the name of a color other than that of the ink. Typically, one is slower in this situation than if the color word and the name of the color coincide.
Trail Making Tests (Reitan and Wolfson, 1986)	Trail Making Tests provide information on visual search, scanning, speed of processing, mental flexibility, and executive functions.

*This noncomprehensive list of validated subjective and objective measures may be used to measure both stroke's potential impact on a participant, as well as a means for measuring change resulting from a BCI-FES intervention in stroke survivors. Included are suggested measures of cognition, affect, motor function and capacity, and activities of daily living (ADLs), all of which cover domains of function potentially impacted by stroke insult*.

Objective and subjective measures of motor capacity and function, measures of task and brain activity, and ADLs (e.g., Barthel Index, Motor Activity Log, etc.), are important metrics to consider when assessing the impact of BCI-FES on users (Simon et al., 2021) (please see Table 4).

## Discussion

BCI-FES systems: (1) have the potential to be significantly more cost-effective than traditional rehabilitations (i.e., naturally modifiable and can be configured to address individuals' needs or environmental constraints such as budget, space or location), (2) provide therapy that supplements, and potentially shortens or replaces conventional poststroke care, and (3) provide rehabilitative therapy that may be superior to present day standards of care, particularly in both the most severely impaired and chronic survivors of stroke. Primary outcome scores (e.g., Action Research Arm Test, Fugl-Meyer Test) following intervention suggest that the present BCI-FES design is able to deliver moderate improvements in UE motor function supported by evidence of similar improvements in several other subjective and objective measures of stroke impact (Song et al., 2014, 2015; Young et al., 2014,a, 2015, 2016; Remsik et al., 2018, 2019, 2021).

The present non-invasive, EEG-based BCI-FES intervention has the potential to improve rehabilitation poststroke over and above the conventional standards of care in use at the present time. Each of the three example participants included herein demonstrated an increased capacity to perform the BCI-FES task accurately (Figure 4 and Table 3) over the course of intervention. Although it may take time for a user to become proficient at the BCI-FES task requirements (i.e., volitional control of the cursor's movement across the screen), nearly all users who are able to understand the instructions are able to use and benefit from the technology. While the features of rehabilitation might differ from person to person, the mechanisms of motor learning and brain-computer interfacing are ubiquitous as they rely on native CNS functioning. The BCI-FES concept is generalized across participants in that the means for using a BCI naturally exist in most all participants, yet the application of the intervention may be personalized. Thus, the BCI-FES intervention presented here allows for clinical translation of BCI-FES technology in a manner that tailors the therapy to the needs and circumstances of specific individuals, thereby providing a basis for personalized, precision medicine.

Recovery of motor function poststroke follows specific neurological patterns and is so far limited in capacity by, among other factors, the individual participant's presenting functional abilities. None the less, BCI devices can be used by participants regardless of severity of stroke lesion or motor impairment and offer a novel tool for delivering treatment options to those who are unable to participate in or benefit from more traditional means of motor rehabilitation. Further, the BCI intervention design presents a means to investigate and improve participant motor performance, beyond the capacity of conventional methods and expectations of care. The portability, adaptability (i.e., gamification) and efficacy of our BCI-FES design are ideally suited to extend windows of care for chronic severely-impaired stroke survivors by providing continued care options beyond traditional clinical settings into, for example, the participant's own home.

The potential therapeutic benefits of using closed-loop neural activity-triggered feedback systems (i.e., BCI-FES) for motor rehabilitation are being investigated in stroke survivors (Feng and Belagaje, 2013). Either FES, which targets specific muscle sets *via* myotic stimulation, or robotic assistance, which acts to replace control of the impaired limb, are able to produce movement of the paretic limb. BCI-FES designs can be configured to drive volitional UE movement rehabilitation and may be tailored to precisely modulate the strength and timing of muscle activity of the recovering motor system (Cho et al., 2011; Stinear, 2016). Recent evidence suggest that BCI-FES is an effective means of delivering treatment beyond traditional clinical windows and BCI-FES designs may be more effective than other existing BCI designs (Biasiucci et al., 2018; Simon et al., 2021). The optimal inclusion of adjuvants and the physical design of a BCI system for stroke motor rehabilitation are yet undefined in the field. Evidence suggests that BCI-FES systems, in combination with traditional PT (e.g., goal-directed motor behaviors, functionally relevant movements as compared to imagined movements, etc.), may facilitate superior improvements in motor recovery by inducing neuroplastic changes in appropriate control circuitry, compared to traditional BCIs, occupational therapies, or robotic rehabilitations (Cervera et al., 2018; Carvalho et al., 2019; Simon et al., 2021). Multimodal feedback from visual, somatosensory, and electro-tactile afference, contingent on EEG-signals related to voluntary movement intent, drives sensorimotor integration and may represent a mechanism, motor learning, responsible for BCI-FES induced motor recovery (Biasiucci et al., 2018). To date, of the various configurations of BCI devices in use for motor recovery, BCI-FES designs have demonstrated superior clinical efficacy (Bai et al., 2020; Simon et al., 2021). In other procedures, FES is used therapeutically to aid voluntary motor function during motor rehabilitation (Merletti et al., 1975; Popovic et al., 2002a,b, 2004, 2009; Popovic, 2014) and contingent integration may be important for successful rehabilitation (Iftime-Nielsen et al., 2012). As demonstrated by Biasiucci and colleagues, in their BCI-FES vs. sham FES experimental design, the inclusion of the FES adjuvant incorporates somatosensory contributions to the BCI user's goal-directed motor plan that are thought to encode afferent information of consequence to the brain facilitating a closed-feedback loop (Biasiucci et al., 2018).

Specifically, in our BCI-FES design, the facilitation of myotic activation contingent on EEG-recorded intent-to-move neuromodulations may foster multimodal—cutaneous, proprioceptive, and visual—afference that aids in enhancing adaptive intra- and interhemispheric network connectivity changes (Remsik et al., 2021). Suitable activation of sensorimotor feedback loops may drive conditioning as well as activity-dependent, Hebbian plasticity (Bach-y-Rita, 1981; Bergquist et al., 2011; Wolpaw, 2012). Whereas our study design (NCT02098265) does not allow us to draw the same conclusions as Biasiucci and colleagues with respect to the precise mechanisms or clinical significance of the FES adjuvant, our results and our BCI-FES device are similar to those of Biasiucci and colleagues (Biasiucci et al., 2018; Remsik et al., 2021). Therefore, it is likely that the clinically-relevant functional gains obtained with the BCI-FES system described here are due to similar strict contingency of BCI-driven FES detailed by Biasiucci and colleagues. However, while Biasiucci and colleagues offer evidence for such a mechanism, the specific sensorimotor substrates and mechanisms that underlie the observed improvements in motor learning remain unknown (Christensen and Grey, 2013).

The present evidence-based protocol delivers meaningful functional improvements; however, additional research is needed to identify the neural circuitry and mechanisms responsible (Biasiucci et al., 2018; Bai et al., 2020). Future research must be directed toward identification and tracking of the genesis and progression of associated neuroplastic changes, and the relative importance of changes in intra- and interhemispheric network connectivity. Continued research into the mechanistic origins of any such neuroplasticity will help improve rehabilitation strategies in order to enable caregivers to provide maximal benefit to patients (Bai et al., 2020; Simon et al., 2021).

### Limitations

While small-scale, observational findings in the use of BCIs for motor rehabilitation have highlighted the promise of this technology for stroke survivors, a standardized BCI-FES intervention schedule and dosing regimen has yet to be recognized for optimal treatment of hemiparesis (Remsik et al., 2016; Bai et al., 2020; Simon et al., 2021). Development of a standard rehabilitation protocol requires large cohort studies and increased monitoring in clinical settings beyond the laboratory.

Heterogeneity in intervention effects may be compounded by the limitations of any given outcome measure (i.e., sensitivity, suitability), and the large variability in location and extent of stroke-induced damage among survivors. As stroke may affect either multiple aspects of one's life, or a stereotyped movement (e.g., hand grasping), it is important to employ a diverse battery of neuropsychological assessments in order to capture adaptive or maladaptive effects that may result from the intervention (Table 4).

#### Design

Adjustments to various components of the BCI-FES intervention design (e.g., more intervention, more frequent intervention, etc.), display enrichment (e.g., enhanced gameplay and graphical presentation), or improvements in functional (i.e., task) relevance (e.g., simple instructed wrist supination and pronation, compared to pouring a virtual glass of liquid into another virtual glass etc.) might further facilitate motor recovery in stroke participants using a BCI-FES with multimodal feedback. Such enhancements to BCI intervention designs might improve participants' engagement, attention, and motivation during the intervention sessions, potentially increasing their neuroplastic effects (Seo et al., 2019). Participants might also benefit from increased monitoring of self-reported fatigue or motivation throughout the intervention sessions. BCI-FES is most effective when participants are actively engaged in the task and, therefore, it may be important to measure changes in engagement due to fatigue, boredom, or other limitations, and lapses in concentration (Seo et al., 2019). Additional research on the effects of these and other considerations not raised here, may help to increase the effectiveness of BCI-FES interventions for UE motor recovery in stroke survivors.

#### Control Features

Although the specific control features that are selected to trigger FES may vary from participant to participant, the common principle between participants is that the features selected derive from EEG frequency bands and cerebral cortical areas known to be associated with sensorimotor processing and voluntary motor output. Thus, the BCI device is adapted to each participant individually, which aids participants with different motor capacities and brain volumes to use the device (Bundy et al., 2012).

#### Dose

Data presented in other work from our laboratory (Young et al., 2014a, 2015, 2016; Song et al., 2015; Mazrooyisebdani et al., 2018; Mohanty et al., 2018; Remsik et al., 2018, 2019, 2021) suggest that a dose of 2-h sessions for up to 30 h with this BCI-FES intervention design is sufficient to positively effect motor recovery in stroke participants. Furthermore, a larger number of runs of this BCI-FES intervention results in greater brain and behavioral changes associated with recovery (Remsik et al., 2018, 2019, 2021). Further research, specifically investigating how behavioral improvements depend on dosage categories (i.e., low, medium, or high) is needed to optimize dosage for specific individuals.

#### Supplemental Stimulation Adjuvants

Incorporating an adjuvant stimulus component (e.g., FES, TDU, haptic feedback, etc.) and multimodal feedback into the BCI intervention design may engender a more dynamic rehabilitative approach (Bach-y-Rita, 1990). Clinical fidelity is thought to depend largely on the sensory feedback that establishes the non-invasive closed-loop system (Biasiucci et al., 2018; Simon et al., 2021). The feedback of the BCI-FES design can help shape the motor efference produced in cerebral cortical motor areas, and when this association remains consistent over time, the brain will adapt. The BCI-FES design presented here can drive that adaptation toward useful recovery of motor function. Inclusion of adjuvants may also pose specific limitations, such as managing consistent placement of the FES electrodes across subjects, across sessions, as well as variations in sensitivity threshold and willingness of participants to receive adjuvants that deliver stimulation. The present BCI-FES design limits participants to simple whole-hand flexion or extension of the fingers (i.e., repeated hand grasping) and some stroke survivors may benefit from practicing different or more complex movements, which the current BCI-FES configuration is not designed to support.

## Conclusion

BCI-FES designs are cost-effective and superior means of delivering poststroke care that are capable of supplementing or partially replacing traditional PT regimens. The BCI-FES is a most promising design for the future of BCI-mediated rehabilitation of stroke. Further improvements in BCI design, such as updating to wireless communication between system components, decreasing system size and cost, as well as gamification and simplification of the user interface, will further minimize costly healthcare supervision and, therefore, will increasingly satisfy requirements of healthcare payers for more cost-effective means to supplement and enhance conventional PT for stroke survivors within and beyond traditional care windows.

The multisensory closed-loop BCI-FES intervention design described here has been shown to be safe and effective for stroke survivors at all timepoints after their initial insult. This intervention design effectively enables users to either continue their recovery beyond standard clinical care windows (i.e., well after their CNS insult—e.g., chronic stroke) or it can function as a supplement to standard of care therapies available within standard clinical care settings (e.g., acute stroke). The closed-loop nature of this BCI-FES design may enhance experience-dependent neuroplasticity (Bach-y-Rita, 1981, 1990; Nudo, 2003a; Wolpaw, 2012), especially in the sensorimotor system, driving neurophysiological changes that promote functional recovery of stroke-impaired UE, regardless of other factors. In this BCI-FES intervention design, FES of the stroke-impaired muscles contingent on participant-generated control features in the recorded EEG signals associated with movement intent elicits subsequent signaling in multiple native sensory (cutaneous, proprioceptive, visuo-motor, etc.) and motor circuits that likely enhance and refine subsequent intent-to-move signals (i.e., motor command signals) and efficacy of subsequent motor behavior. This work represents a first step toward clinical translation of a standardized design for BCI-FES interventions.

## Data Availability Statement

The original contributions presented in the study are included in the article/supplementary files, further inquiries can be directed to the corresponding author/s.

## Ethics Statement

The studies involving human participants were reviewed and approved by University of Wisconsin Health Sciences Institutional Review Board (Study ID 2015-0469). The patients/participants provided their written informed consent to participate in this study.

## Author Contributions

AR, LW, and SG were involved in all aspects of this manuscript. PK and KG contributed to manuscript writing and editing as well as intellectual content. VN was involved in participant recruitment, data collection, manuscript editing and intellectual content. KC, JW, and VP are co-PIs and were involved in all aspects of the study as well as manuscript conception, design, editing and intellectual content. All authors contributed to the writing of this manuscript. All authors contributed to the article and approved the submitted version.

## Funding

This work was supported by NIH grants 1R01NS105 646-01A1, RC1MH090912-01, T32GM008692, UL1TR000427, K23NS086852, T32EB011434, R01EB000856-06, R01EB009103-01, and R01EB009103-01 and by the DARPA RCI Program (MTO) N66001-12-C-4025 and HIST Program (MTO) N66001-11-1-4013. Additional funding was also provided through a Coulter Translational Research Award, the American Heart Association Grant 1T32EB011434-01A1, AHA Innovative Research Award—National (Marcus Foundation) 15IRG22760009, AHA Midwest Grant in Aid Award 15GRNT25780033, the Foundation of ASNR, UW Milwaukee-Madison Intercampus Grants, the UW Graduate School, and by Shapiro Foundation Grants.

## Conflict of Interest

JW is a scientific board member and has stock interests in NeuroOne Medical Inc., a company developing next generation epilepsy monitoring devices. JW also has an equity interest in NeuroNexus technology Inc., a company that supplies electrophysiology equipment and multichannel probes to the neuroscience research community. JW also has an equity interest in Neuraworx Medical Technologies Inc., a company developing non-invasive neuromodulation devices for treating cognitive impairments. None of these associations are directly relevant to the work presented in this manuscript. The remaining authors declare that the research was conducted in the absence of any commercial or financial relationships that could be construed as a potential conflict of interest.

## Publisher's Note

All claims expressed in this article are solely those of the authors and do not necessarily represent those of their affiliated organizations, or those of the publisher, the editors and the reviewers. Any product that may be evaluated in this article, or claim that may be made by its manufacturer, is not guaranteed or endorsed by the publisher.
